# Development and validation of an infant facial skin assessment tool: a prospective observational study

**DOI:** 10.1186/s12887-022-03691-7

**Published:** 2022-10-25

**Authors:** Manami Matsubara, Megumi Haruna, Kaori Yonezawa, Moeri Yokoyama, Emi Tahara-Sasagawa, Naoko Hikita, Yoshie Nakamura, Yoko Mizuno, Hiromi Sanada, Nao Tamai, Masatoshi Abe, Kosuke Kashiwabara

**Affiliations:** 1 Department of Midwifery and Women’s Health, Division of Health Sciences and Nursing, Graduate School of Medicine, The University of Tokyo, 7-3-1 Hongo, Bunkyo-ku, Tokyo, 113-0033 Japan; 2 The Global Nursing Research Center, Graduate School of Medicine, The University of Tokyo, 7-3-1 Hongo, Bunkyo-ku, Tokyo, 113-0033 Japan; 3 Tohto Bunkyo Hospital Pediatrics, Tokyo, Japan; 4 Department of Gerontological Nursing/Wound Care Management, Graduate School of Medicine, The University of Tokyo, Tokyo, Japan; 5 Department of Imaging Nursing Science, Graduate School of Medicine, The University of Tokyo, Tokyo, Japan; 6 Sapporo Skin Clinic, Sapporo, Hokkaido Japan; 7 The University of Tokyo Hospital, Tokyo, Japan

**Keywords:** Skin, Assessment, Tool, Caregiver, Infants

## Abstract

**Background:**

Severe infant eczema on the face should be treated early because it may lead to allergic diseases in the future. However, caregivers find it difficult to assess. A visual tool for caregivers is needed to easily determine infants’ facial skin condition severity based on the tool’s scores. We developed an infant facial skin assessment tool (IFSAT) and evaluated its reliability and validity.

**Methods:**

The IFSAT draft was developed based on results of a previous literature review and qualitative sketch. Panels including medical professionals and a caregiver checked the draft’s content and face validity, and the IFSAT was finalized. To test the IFSAT’s reliability and validity, caregivers and one-month-old infants were recruited. Two scoring methods were additionally created based on the relation between the items and cure period. The relationships between scores and cure period, and the ability to predict whether the infant needed medical treatment were examined by each scoring method. For the predictive validity, scores for infants requiring medical treatment and those for infants who did not were also compared. For the intra-examiner reliability analysis, two pediatricians rated the scores separately twice using photographs. Inter-rater reliabilities were analyzed among pediatricians, nurses, and caregivers.

**Results:**

Altogether, 113 infant-caregiver pairs participated in the testing phase. Of the two scoring methods created (versions 1 and 2), pediatricians’ and caregivers’ scores using versions 1 and 2 were related to the cure period. These scores predict whether the infant needed medical treatment. We then selected version 2 based on the medical professionals’ opinions. The scores of caregivers of infants requiring medical treatment were higher than those of caregivers of infants not requiring treatment (*p* < 0.001). The intraclass correlation coefficient (ICC) of intra-examiner reliability was 0.87. The ICC of inter-rater reliabilities between pediatricians’ and caregivers’ scores and between nurses’ and caregivers’ scores were 0.66, and 0.66, respectively.

**Conclusions:**

The proposed IFSAT may be used to assess whether infants need medical treatment and whether to extend the cure period. The tool’s reliability and validity were confirmed.

**Supplementary Information:**

The online version contains supplementary material available at 10.1186/s12887-022-03691-7.

## Background

Infant eczema may lead to allergic diseases in the future. The presence of eczema is associated with disrupted skin barrier function which protects the body from external stimuli. Thus, it becomes easier to become sensitize to allergens [1]. According to a previous study, eczema in infants that occurred within the first 1–2 months after birth may possibly progress to severe skin conditions related to food allergies at 3 years of age [2]. Infant eczema frequently appears on the infants’ faces. The infants’ faces are exposed to the air and may touch allergens easily when infant eczema appears. Thus, infant eczema on the face should be paid special attention. Although most cases of eczema on the face in infants, including seborrheic dermatitis and infant acne, can cure quickly, some require a longer cure period. If the eczema appears longer, the eczema may continue to disrupt the skin barrier for a longer time. Therefore, infants’ skin conditions that takes longer to cure should be recognized as severe cases. Moreover, some infants need medical treatment on their face. When the infants’ skin condition is needed medical treatment, the skin barrier may also be damaged. Thus, such a condition, which needed medical treatment, should be also recognized as severe. To minimize damage to the skin barrier function, such severe eczema in infant should be treated early. To treat severe infant eczema early, caregivers must first determine if the infant eczema is severe at home or in other settings where they cannot immediately consult a medical professional. However, many caregivers find it difficult to assess an infant's facial skin condition without optimal knowledge of which infant skin conditions are more serious.

One solution to the above issues is to provide a visual tool for caregivers to determine the severity of their infants’ facial skin condition easily based on the scores of the tool, whether medical treatment is needed, or whether the cure period would be longer. However, a previous review revealed that existing tools do not focus on facial skin conditions; some scales focus on diaper rash and the skin condition of the whole body [3]. Thus, there is a need to develop a new assessment tool specific to infant eczema on the face and designed for caregiver use.

The purpose of this study was to develop a new infant facial skin assessment tool (IFSAT) that enables caregivers to assess the severity of eczema on the face of infants and to examine its reliability and validity. In this study, severe eczema on the face of infants is defined as the condition that may take days to be cured or the condition requiring medical treatment according to a pediatrician evaluation. In the development phase, a caregiver evaluates whether the tool is easy to fill in and use.

## Methods

This study included the following two phases: “development of the IFSAT” and “testing to determine its reliability and validity”.

### Development of the IFSAT

First, a qualitative sketch was conducted to evaluate the assessment items for the symptoms and divide the infant’s facial areas, where each item was likely to appear. According to the qualitative sketch method [4], we made sketches with reference to photographs of infants’ facial skin problems and summarized assessments from them. These photographs were taken in a previous study, which was targeted for infants 1 month age [5]. Then, descriptions of symptoms of eczema in infant and their preferred areas were extracted from the summaries. The draft of the IFSAT was designed based on the results of the qualitative sketch and a previous review. The review searched 429 articles and summarized the observational items of two articles on facial skin problems in infants that met the inclusion criteria. The results have already been submitted [3].

Second, to examine the content and face validity of the draft, six medical professionals (one pediatrician, two dermatologists, one public health nurse, one midwife, and one nurse certified in wound, ostomy, and continence nursing) and one caregiver were recruited. They provided informed consent for participation. The medical professionals and caregiver comprising the panel for the development phase did not participate in the validation testing. The researcher (MM) asked them to review the draft in terms of the types of assessment items (e.g., Did the draft include enough items about the symptoms?), division of facial areas (e.g., Was the division of facial areas adequate?), and expression of assessment items (e.g., Was the expression adequate to understand the meaning of contents? Was the draft easy to fill in?) in semi-structured interviews. After conducting one-on-one interviews and gathering tentative opinions about the draft, we discussed each opinion with our research team (MM, KY, MY and MH) and decided on which opinions would be used to revise the draft. Then, the draft was revised based on panel feedback and the IFSAT was created. If two completely opposite opinions came up in the same question, we referred to the opinion expressed by more experts. Finally, the pediatricians and nurses who were the staff of the hospital where the validation testing was conducted assessed some infants’ skin conditions using the tool during 1-month health check-ups of infants to assess its contents and expression.

### Testing to determine reliability and validity

In the testing phase, we assessed inter-rater and intra-examiner reliability as a test of reliability. We also assessed predictive and concurrent validity as a test of validity. This prospective, observational study was conducted at regular health checkups of 1-month old infants at a hospital in Tokyo from July 2018 to March 2019. Two pediatricians were in charge of the 1-month health checkups at that hospital in Tokyo. Caregivers of 1-month-old infants provided informed consent for participation in the study. The inclusion criterion for 1-month infants was a gestational age ≥ 37 weeks. Infants whose caregivers could not read and write Japanese were excluded. As this study was conducted at a single site, there may have been selection bias due to the demographics of the participants. According to the COSMIN Study Design checklist for Patient-reported outcome measurement instruments, the minimum sample size for this study was 100 infant-caregiver pairs [6].

At the 1-month health checkup, an infant’s caregiver filled in the tool while observing their infant’s face during the waiting time. Additionally, one of the two pediatricians and one of the five nurses who were in charge of the 1-month health checkup of the infant filled in the IFSAT during medical checkups. To control desirability bias, we set that the caregivers did not see the instrument filled out by the pediatricians and nurses. The caregivers provided information about their infants’ and their characteristics, such as demographic data, using a questionnaire.

### Decision of the method of scoring and the predictive validity

Pediatricians also evaluated whether the infants’ facial conditions were needed medical treatment. In this study, if pediatricians evaluated that the infant may need medical treatment, it was considered that they thought the eczema on the face of infants was severe and wanted to treat the infant’s skin (hereinafter referred to as infants who needed medical treatment). The results of this evaluation by the pediatricians were used as a proxy for the “gold standards,” because pediatricians often treat the infants’ facial skin and determine whether the skin condition needs consultation by a dermatologist. After the 1-month health checkup, the caregivers reported the date on which their infants’ skin problems disappeared. A researcher sent an email to each caregiver for a maximum of 5 weeks after their infant’s regular checkup. The interval between the 1-month health checkup and the disappearance of the infant’s skin problems was considered as the “cure period.”

To develop a tool in which scores reflect the difficulty in curing, two versions of the scoring method (version1 and version 2) were additionally created by combining some facial areas and/or by rating items based on the relationship between items and the cure period. The relationship between each of the scoring methods’ scores (pediatricians’ scores, nurses’ scores, and caregivers’ scores) and the cure period were then examined. Additionally, also the ability to predict whether the infant needed medical treatment were examined by each scoring method. Then, one of the methods was chosen. The caregivers’ cut-off scores were calculated based on the ability to predict whether the infant needed medical treatment. Then, for predictive validity, we checked whether the pediatricians’, nurses’, and caregivers’ scores of the infants who needed medical treatment is higher than those of infants who did not need medical treatment.

### Intra-examiner reliability

The researcher took photographs of the infants at 1-month checkups. Then, two pediatrician who checked the infant’s skin condition at the 1-month health checkup completed the assessment tools using the 10 infants’ photographs each after health checkups (Time 1) and rechecked the assessment tools using the same photographs after more than 2 weeks from Time 1 (Time 2). Then, the Time 1 and Time 2 scores were compared for intra-examiner reliability.

### Inter-rater reliability

The scores of pediatricians, nurses, and caregivers from the assessment tool at the 1-month health checkups were compared. Additionally, the agreement rate between the two pediatricians who worked in different shifts regarding whether infants needed medical treatment was also calculated. Data regarding the need for consultation were collected using 20 infants’ photographs taken at 1-month checkups.

### Concurrent validity

Correlations between the scores of the pediatricians, nurses, or caregivers and skin barrier functions were analyzed. The examination of skin barrier functions included transepidermal water loss (TEWL, Tewameter®TM300, Courage + Khazaka), stratum corneum hydration (SCH, Corneometer®CM825, Courage + Khazaka), and sebum (Sebumeter®SM815; Courage + Khazaka) on the forehead.

**Fig. 1:**
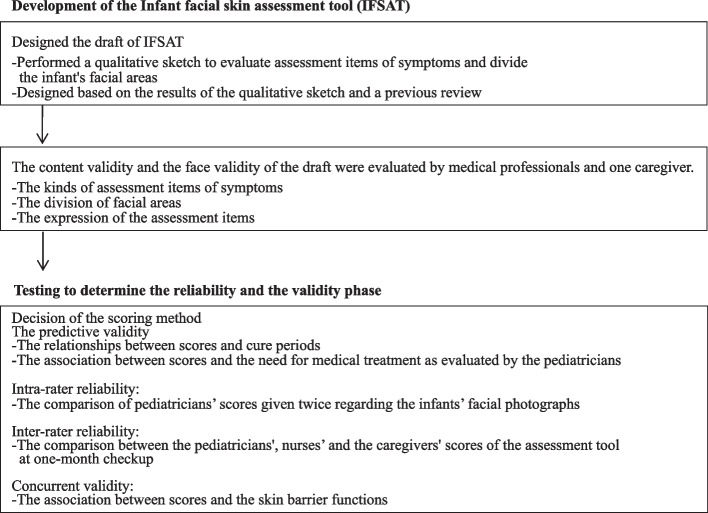
Flowchart of study protocol

Figure 1 shows the flowchart of this study protocol.

### Statistical analyses

Demographic data were calculated as medians or percentages. The relationships between the scores of the pediatricians or nurses or caregivers among the groups and the cure periods were compared using the Jonckheere test. On analyzing the duration of cure periods, infants who used dermatologic agents after birth were excluded because the cure period would be affected by these agents. Additionally, a receiver operating characteristic (ROC) analysis was performed to compare the area under the curve (AUC) of each version of the scoring system. The cut-off scores based on the need for consultation were calculated using the Youden index. Relationships between pediatricians’ or nurses’ or caregivers’ scores and whether infants needed contact with a medical staff were calculated using the t-test and Mann–Whitney U test. Intra-rater reliability was examined using intraclass correlation coefficients (ICC). Inter-rater reliability among pediatricians, nurses and caregivers were evaluated using ICC. The agreement rate regarding the need for medical treatment between the two pediatricians was calculated as a percentage. Correlations between the scores and skin barrier functions were examined using Spearman’s rank correlation coefficient (r). Significance was set at *p* < 0.05. All analyses were conducted using SPSS version 27.0 for Windows (IBM Corp., Armonk, NY, USA). Additional file 1 was draw using Excel® 2019 ® (Microsoft Corp., Redmond, USA).

## Results

### Development of the IFSAT

A qualitative sketch was made using the facial photographs of 20 infants. Redness, papules, dryness, and yellow scaling were extracted as assessment items for the symptoms. As erythema and purpura could not be distinguished in the photographs, they were extracted as redness. The previous review also extracted dryness and desquamation, papules, and erythema as symptoms of infantile eczema, and erythema, dryness and desquamation, and scaling as symptoms of seborrheic eczema [3]. Thus, the draft of the assessment tool included erythema, papules, dryness, and yellow scaling, as these items were matched between the results of the qualitative sketch and the results of a previous review. Additionally, the draft included purpura to strictly assess redness. Considering the appearance of these items based on this result and the common site of occurrence of skin conditions, the infant’s face was divided into 11 areas, which were as follows: right forehead, left forehead, brows, right eye, left eye, nose, right cheek, left cheek, mouth and jaw, right ear, and left ear. Then, the medical professionals and the caregiver checked the assessment items with respect to eczema in infant, division of facial areas, and expression of each assessment item for content and face validity. Based on their feedback, purpura was excluded, because purpura was confused with pigmentation. Yellow scaling was combined with exudate. Additionally, the number of facial areas was reduced, because all other interviewees except one medical professional mentioned that the number of areas should be reduced. The presence or absence of items of all facial areas were listed in one table before evaluating the validity of the draft. However, based on their opinions, we modified the structure of IFSAT in order to write the presence or absence of items near each facial area.

Finally, pediatricians and nurses checked the content and face validity of the tool at the 1-month health checkup, and the IFSAT was finalized.

**Fig. 2:**
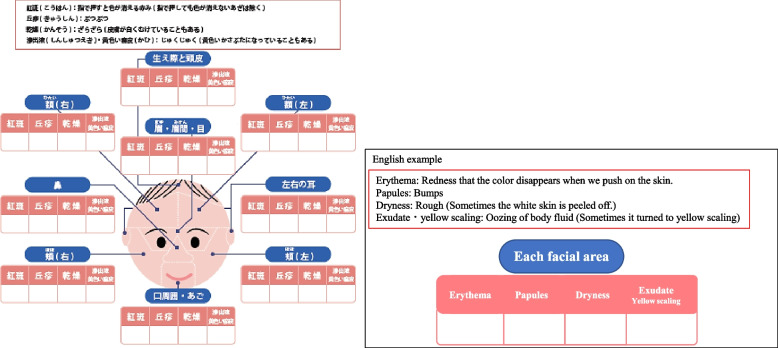
IFSAT

Figure 2 shows the IFSAT. The tool includes four items of skin problems (erythema, papules, dryness, and exudate/yellow scaling) and nine facial areas (scalp and hairline, right forehead, left forehead, eyebrows/between the eyebrows/eyes, nose, right cheek, left cheek, around the mouth/jaw, and ears).

### Testing to determine the reliability and validity

A total of 122 infant caregivers were recruited, and 113 infant-caregiver pairs participated in and completed the research at the 1-month health checkups. However, 21 infant-caregiver pairs dropped out within 4 weeks after the 1-month health checkups, and 92 infant-caregiver pairs completed the observation period (cure period). On analyzing the duration of the cure periods, 13 infants who used dermatologic agents after birth were excluded. Thus, 79 infant-caregiver pairs were included in the analysis of the duration of the cure periods.

Table 1 shows the characteristics of the participants. Twenty-nine infants (25.7%) had a family history of atopic dermatitis. Among the caregivers, 109 (96.5%) were mothers, and one (0.9%) was a father. One pediatrician had more than 10 years of clinical experience, while the other had a clinical experience of more than 20 years.

**Table 1:** Participants' characteristics in the validation test

	n (%) or Median (IQR)^a^
*Infants (n* = *113)*	
Sex	
Male	53 (46.9)
Female	60 (53.1)
Age (days)	32 (31–35)
Birth weight (g)	3070 (2897–3229)
Gestational age (weeks)	39 (39–40)
Family history of atopic dermatitis	29 (25.7)
IFSAT scores of infants who was prescribed at one-month checkups (*n* = 9)	
Pediatrician	16.00 (13.50–27.50)
Nurses	15.00 (12.50–16.50)
Caregivers	14.00 (11.50–17.50)
*Infants' caregivers’ Attribution*	
Mother	109 (96.5)
Father	1 (0.9)
Both parents	3 (2.7)
*Pediatricians (n* = *2)*	
Clinical experience	
> 10 years	1 (50)
> 20 years	1 (50)

^a^ *IQR* Interquartile Range (25th percentile – 75th percentile)

### Decision of the scoring method and the predictive validity

The items erythema_left forehead, erythema_right forehead, papules_left forehead, papules_right forehead, papules_left cheek, papules_right cheek, and papules_mouth showed longer cure periods (see Additional file 1 Fig S1–5). Additionally, the total scores of dryness and exudate were determined because these skin problems were more likely to have a longer cure period. Moreover, two versions of the scoring system were created. In one version (version 1), the scores of items erythema_left forehead, erythema_right forehead, papules_left forehead, papules right forehead, papules_left cheek, papules_right cheek, papules_mouth, dryness, and exudate were doubled. In the other version (version 2), two facial areas (right forehead and left forehead) were combined to one area (forehead), and the scores of the items (erythema_left forehead, erythema_right forehead, papules_left forehead, papules right forehead, papules_left cheek, papules_right cheek, papules_mouth, dryness, and exudate) were doubled.

Moreover, the relationships between the duration of the cure period and the total score of each version or the scores of the original tool (not rating scores) were compared (Table 2). The infants were divided into four groups according to the duration (days) of the cure periods, which were as follows: 0–7, 8–14, 15–28, and > 28 days (the infants’ skin problems did not disappear within 4 weeks after the 1-month health checkup). The results of the analysis of the relationships between the tool’s scores and the cure period showed that both pediatricians’ and caregivers’ scores among the original, version 1, and version 2 tools were related to the cure period. Regarding the need for medical treatment, the AUC values of all the scoring methods were over 0.80. Finally, we chose version 2 because the medical professionals preferred a smaller number of facial areas. The cutoff value based on the caregivers scores’ Youden index was 8 (caregivers scores; sensitivity, 1.00; specificity, 0.55; Youden Index, 0.55).

**Table 2:** The relationships between cure periods and scores and AUC based on “the need for medical treatment”

Version	Rater	Cure period (days)					AUC^f^
		0–7 (*n* = 15)	8–14 (*n* = 19)	15–28 (*n* = 20)	over 28 (*n* = 25)	*P* value^d^	(*n* = 113)
		Median (IQR^c^)	Median (IQR^c^)	Median (IQR^c^)	Median (IQR^c^)		
Original	Pediatricians	1.0 (0.00–2.00)	3.0 (1.00–7.00)	2.5 (0.25–5.50)	4.0 (2.00–7.50)	0.03^e^	0.84
	Nurses	2.0 (0.00–5.00)	2.0 (1.00–4.00)	2.5 (1.00–6.00)	3.0 (2.00–5.50)	0.07	0.85
	Caregivers	3.0 (2.00–4.00)	4.0 (3.00–6.00)	4.0 (3.00–6.00)	5.0 (3.00–8.00)	0.03^e^	0.89
Version 1^a^	Pediatricians	1.0 (0.00–4.00)	6.0 (2.00–10.00)	5.0 (0.50–9.50)	7.0 (4.00–13.50)	0.02^e^	0.84
	Nurses	2.0 (0.00–7.00)	4.0 (2.00–6.00)	4.5 (2.00–11.50)	5.0 (2.00–8.50)	0.06	0.85
	Caregivers	5.0 (4.00–7.00)	8.0 (6.00–11.00)	8.0 (6.00–9.75)	9.0 (5.00–12.50)	0.03^e^	0.86
Version 2^b^	Pediatricians	1.0 (0.00–4.00)	6.0 (2.00–10.00)	4.0 (0.50–8.00)	6.0 (3.00–10.50)	0.03^e^	0.86
	Nurses	2.0 (0.00–7.00)	4.0 (0.00–6.00)	4.0 (2.00–7.75)	5.0 (2.00–8.50)	0.09	0.86
	Caregivers	5.0 (4.00–7.00)	8.0 (6.00–11.00)	7.5 (6.00–9.75)	9.0 (5.00–11.00)	0.04^e^	0.87

^a^ Version 1 scoring system (erythema_left forehead*2, erythema_right forehead*2, papules_left forehead*2, papules_right forehead*2, papules_left cheek*2, papules_right cheek*2, papules_mouth*2, dryness total score*2, exudate total score*2)

^b^ Version 2 scoring system (To combine the left and the right forehead, erythema_forehead*2, papules_forehead*2, papules_left cheek*2, papules_right cheek*2, papules_mouth*2, dryness total score*2, exudate total score*2)

^c^ *IQR* Interquartile range (25th percentile – 75th percentile)

^d^ *p* value was shown when Jonckheere test was performed

^e^ *p* value < 0.05

^f^ Area under curve when a receiver operating characteristic (ROC) analysis was performed

IFSAT scoring method:

1. To combine the left and the right forehead. If there were no symptoms on either the left or right forehead, the infant was considered not to have symptoms. Otherwise, symptoms were present.
2. If erythema on the forehead, papules on the forehead, papules on the left cheek, papules on the right cheek, or papules on the mouth are seen, the score should be 2 points for each. For other items, the score should be 1 point.
3. Calculate the total score for each symptom. After doubling the total score each for dryness and exudate, the total score for each symptom is added together to obtain the IFSAT score.

Table 3 shows the scores of the IFSAT by raters based on the “need for medical treatment” or “no need for medical treatment,” as evaluated by the pediatricians (a proxy of the “gold standard”). In this study, a total of 11 infants were indicated by pediatricians to need medical treatment. The medians of the pediatricians’, nurses’, and caregivers’ scores for the 11 infants who need medical treatment were significantly higher than those for infants who did not need medical treatment (*p* < 0.001, *p* < 0.001, and *p* < 0.001, respectively).

**Table 3:** The scores of the assessment tool by raters depended on the “need for” and “no need for medical treatment” assessed by the pediatricians (*n* = 113)

	Need (*n* = 11)^a^	No need (*n* = 102)^b^	*P* value^d^
	Median (IQR^c^)	Median (IQR^c^)	
Pediatricians	16.00 (8.00–24.00)	4.00 (2.00–9.00)	< 0.001^e^
Nurses	14.00 (8.00–17.00)	4.00 (2.00–7.25)	< 0.001^e^
Caregivers	14.00 (10.00–16.00)	7.00 (4.75–10.00)	< 0.001^e^

^a^ Infants who needed medical treatment

^b^ Infants who did not need medical treatment

^c^ *IQR* Interquartile range (25th percentile – 75th percentile)

^d^ Mann–Whitney U test

^e^ *p* < 0.05

### Intra-examiner reliability

The ICC between the pediatricians’ assessments at the two time points for intra-examiner reliability was 0.87 (95% confidence interval [95% CI], 0.71–0.95).

### Inter-rater reliability

The ICC between pediatricians’ and caregivers’ scores and between nurses’ and caregivers’ scores were 0.66 (95% CI, 0.52–0.76) and 0.66 (95%CI, 0.43–0.79), respectively. The agreement rate between the two pediatricians regarding whether infants needed medical treatment was 100%.

### Concurrent validity

The median and interquartile ranges (IQR, 25th–75th percentile) of temperature and humidity of the room where the skin barrier functions were measured were 28.0 °C (IQR, 26.9–29.0) and 54.4% (IQR, 42.4–58.7), respectively. Correlations were analyzed between pediatricians’, nurses’, or caregivers’ scores and each skin barrier function. TEWL, SCH, and sebum were not significantly associated with the scores, because each correlation coefficient was less than |0.4| (Table 4).

**Table 4:** Correlation between raters’scores and skin barrier functions (*n* = 113)^a^

	TEWL^b^	SCH^c^	Sebum
Pediatricians	0.03	-0.03	0.06
Nurses	0.00	-0.12	0.07
Caregivers	-0.03	-0.05	-0.04

^a^ Spearman’s rank correlation coefficient

^b^ Transepidermal water loss

^c^ Stratum corneum hydration

## Discussion

In this study, we developed the IFSAT based on literature review and a qualitative sketch. Then, we revised the tool according to the medical professionals’ opinions. In the testing phase, we were able to meet the sample size and validated the IFSAT that caregivers can use to assess their infants’ facial skin condition. Additionally, we suggest the version of the scoring method based on the results of the analysis of the relationships between scores and cure periods and the results of the ROC analysis.

In this study, severe infant eczema on the face was defined as the condition that may take days to be cured or the condition that a pediatrician think requires medical treatment. In these conditions, the skin barrier function is considered to have disrupted. Then, the infant prognosis is likely to be affected. According to a previous study, parents have low awareness of the presence of seborrheic dermatitis, which is a type of infant eczema [7]. When the infant’s parents are not aware of the presence of severe infant eczema, the medical treatment may be delayed, and impaired skin barrier function may worsen. Infant eczema is likely to appear after discharge and cannot be continuously observed by the medical staff. Therefore, parents should be able to recognize severe eczema at first. We suggested that caregivers’ IFSAT score might be able to predict the need for medical treatment. This result means that based on IFSAT scores, caregivers may be able to assess whether the infant eczema on the face is severe and whether pediatricians wanted to treat the infant’s skin. Regarding this point, our tool is helpful for caregivers to assess the severity of the infant eczema on the face.

We found that the intra-examiner reliability of the pediatricians was high. This may indicate that the assessments made using this tool are stable. On reliability testing, we found a substantial correlation of the scores among pediatricians, nurses, and caregivers. However, we found that many of the scales that medical staff or patients usually use to assess their infants’ skin has moderate to high correlation coefficients [8, 9]. Thus, the correlations of scores among pediatricians, nurses, and caregivers were within the appropriate range. Therefore, from the results of the intra- and inter-rater reliability tests, caregivers may be able to assess the severity of infants’ facial skin condition, similar to pediatricians and nurses. This suggestion may be useful to caregivers when they are making decisions in their own home, where they cannot immediately consult a medical professional, regarding their infants' need for medical treatment. Additionally, pediatricians and nurses may be able to use the cutoff value based on the caregivers scores because of appropriate inter-rater reliability.

Furthermore, the scores of the IFSAT, which the caregivers filled in, were related to the length of the cure period. Infants’ facial skin condition that takes a long time to cure may indicate that the skin barrier is impaired for a long time. According to this result, caregivers may be able to assess whether the skin condition would require a longer cure period. Furthermore, we should prevent the development of such facial skin condition by doing skin care. However, only a few studies on skin care targeting such facial skin condition because this condition was difficult to evaluate. Therefore, the method of skin care suitable for preventing such facial skin condition is unknown. In future research, IFSAT can be used to determine whether the participants’ skin condition may take a long time to cure or not.

In this study, no significant correlation was found between the scores and the level of skin barrier function. Two factors can explain this finding. First, the levels of skin barrier function may only correlate with the severity of eczema in infants in the measurement area. The present tool includes all facial areas, including the cheek and mouth. Thus, we assessed the severity of not only the conditions affecting the forehead (where we measured the levels of skin barrier function) but also eczema throughout the face in infant using the tool. However, the levels of skin barrier function represent only the skin condition of the measurement area. Therefore, severe facial skin problems involving the whole face may not be accurately represented by the skin barrier functions of a part of the face. Second, we could not measure the infants’ skin barrier function in the same environment because we conducted research during regular health checkups in all seasons. Thus, temperatures and humidity greatly varied. The levels of skin barrier function are easily affected by temperature and humidity because TEWL and SCH are water-related parameters, and sebum secretion is affected by sweating. Thus, the results of this study might be affected by environmental factors.

This study has some limitations. Firstly, our study may not have had an adequate sample size, as only data from 79 infants could be used for the analysis of cure periods. Second, only the symptoms of 20 infants were used during the qualitative sketches. Therefore, some rare symptoms may have been missed. However, our validation test results showed no symptoms other than those extracted from the qualitative sketches. Thus, this limitation does not have a great impact on the purpose of this scale, which aims to encourage caregivers to seek medical treatment once infants show certain severe symptoms. A replication study should be conducted in the future to confirm the symptoms. Finally, considering the burden on the caregivers, we tracked the cure period up to 35 days after the 1-month regular health checkups. Thus, we could not collect data regarding the duration of the cure periods for some infants whose skin conditions were not cured. Therefore, we cannot suggest a correlation between the scores and duration of the cure periods.

Despite its limitation, our assessment tool for the facial skin condition of 1-month-old infants may predict the “need for medical treatment and the duration of the cure period.

## Conclusions

The proposed IFSAT may be used to assess whether 1-month-old infants need medical treatment and whether the cure period will be extended. The reliability and validity of this tool were confirmed. A reliable skin assessment tool will shorten the duration of dermatitis in infants. In the future, a validity analysis of the proposed tool in older infants is warranted.

## Supplementary Information

**Additional file 1: Fig S1.** Relationship between number of infants who had any skin problems and the cure period (days). **Fig S2.** Relationship between number of infants who had erythema and the cure period (days). (*n*=24). **Fig S3.** Relationship between number of infants who had papules and the cure period (days). (*n*=53). **Fig S4.** Relationship between number of infants who had dryness and the cure period (days). (*n*=21). **Fig S5.** Relationship between number of infants who had exudate and the cure period (days). (*n*=10).

## Data Availability

The datasets used and analysed during the current study are available from the corresponding author on reasonable request.

## Abbreviations

IFSAT: Infant facial skin assessment tool
AUC: Area under the curve
CI: Confidence interval
ROC: Receiver operating characteristic
SCH: Stratum corneum hydration
TEWL: Transepidermal water loss
