# Supplementary material for: Development and validation of an infant facial skin assessment tool: a prospective observational study
Source: BMC Pediatr. 2022 Oct 25;22:614. doi: 10.1186/s12887-022-03691-7 (PMC9598006; doi:10.1186/s12887-022-03691-7)
Supplement: Supplementary file 1 — Additional file 1: Fig S1. Relationship between number of infants who had any skin problems and the cure period (days). Fig S2. Relationship between number of infants who had erythema and the cure period (days). (n=24). Fig S3. Relationship between number of infants who had papules and the cure period (days). (n=53). Fig S4. Relationship between number of infants who had dryness and the cure period (days). (n=21). Fig S5. Relationship between number of infants who had exudate and the cure period (days). (n=10). [file 12887_2022_3691_MOESM1_ESM.pdf]

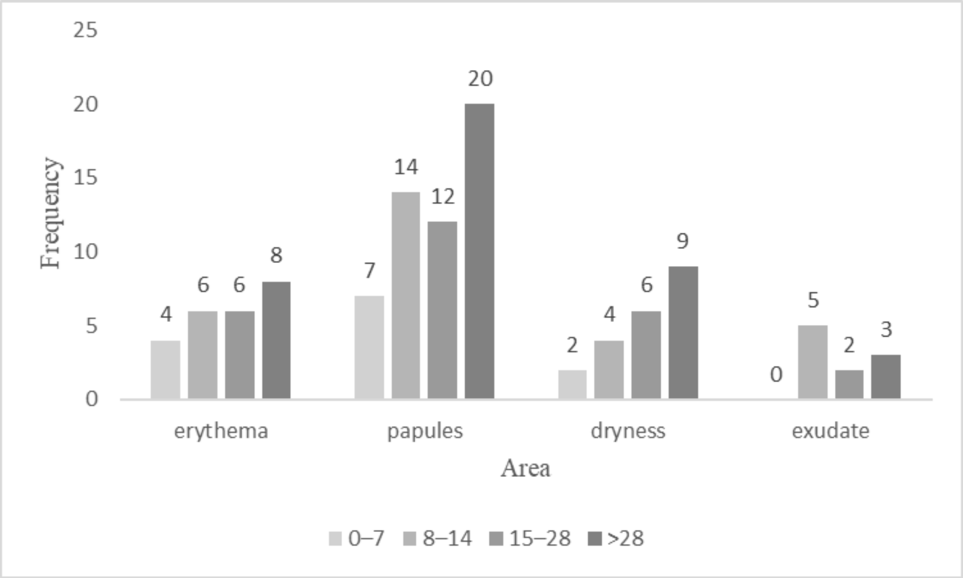

Fig S1: Relationship between number of infants who had any of skin problems and the cure period (days).

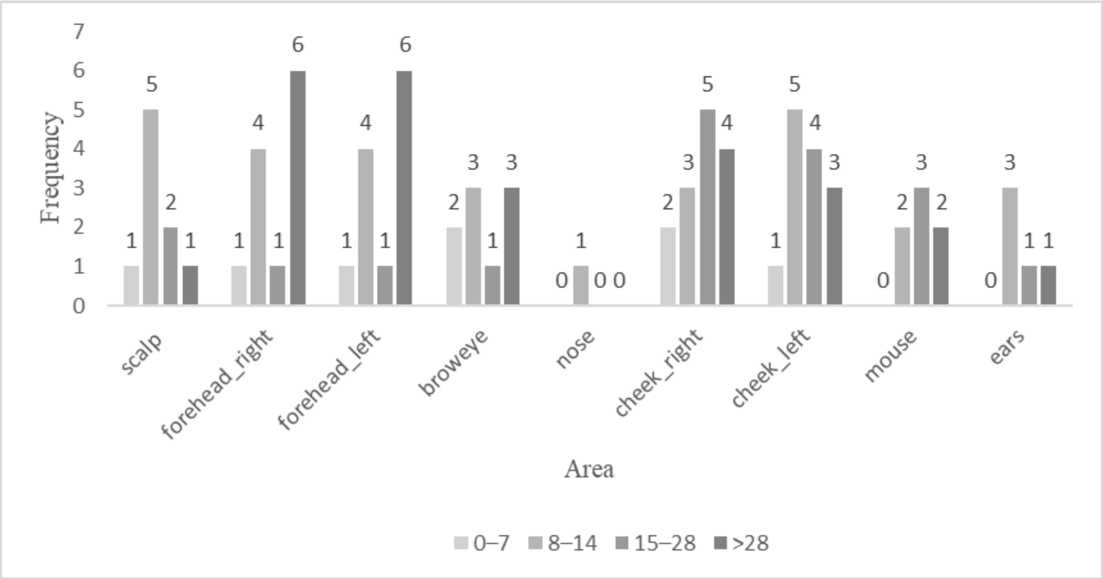

Fig S2: Relationship between number of infants who had erythema and the cure period (days). (n=24)

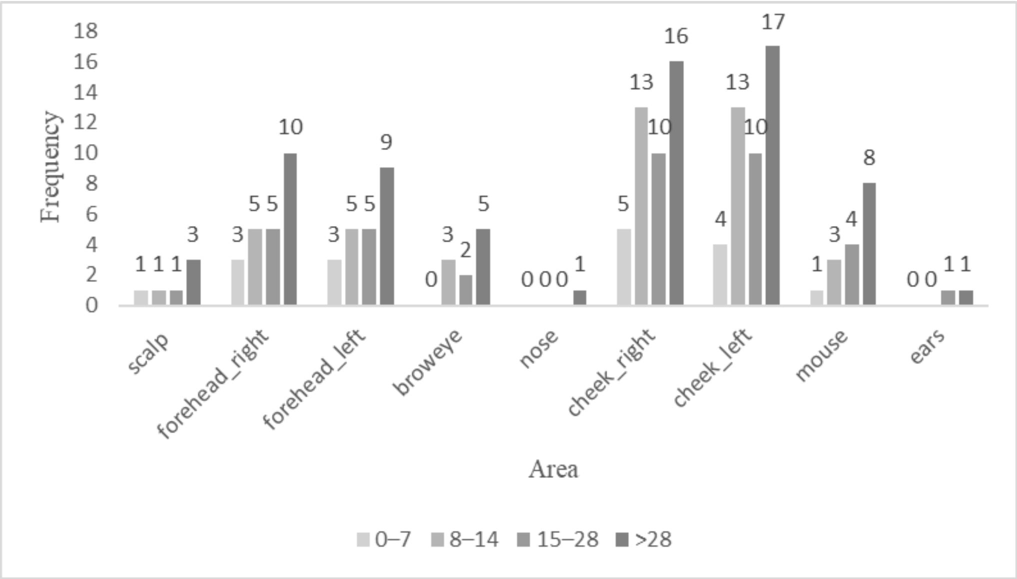

Fig S3: Relationship between number of infants who had papules and the cure period (days). (n=53)

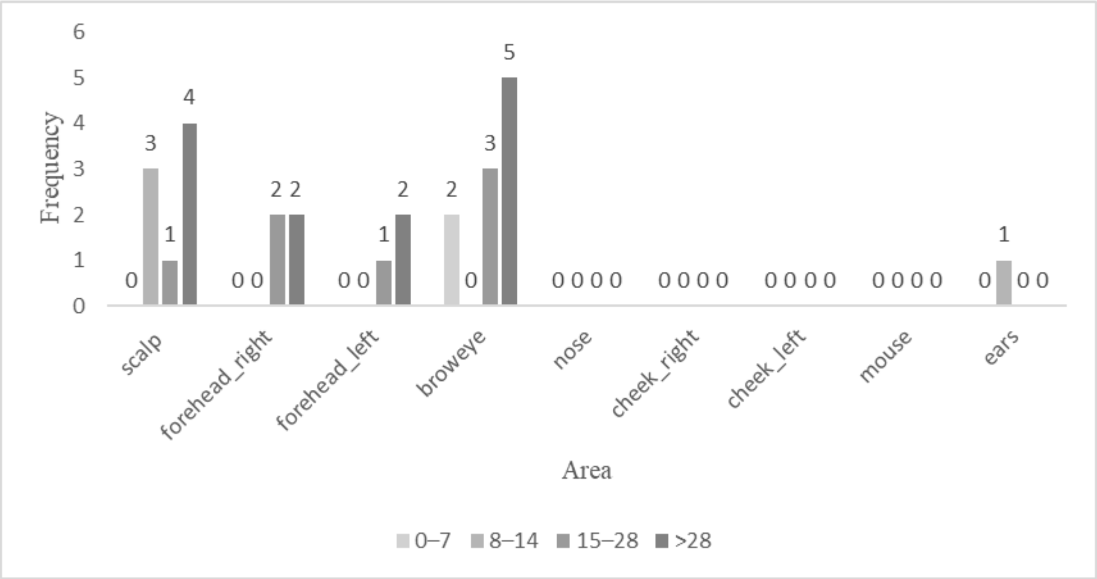

Fig S4: Relationship between number of infants who had dryness and the cure period (days). (n=21)

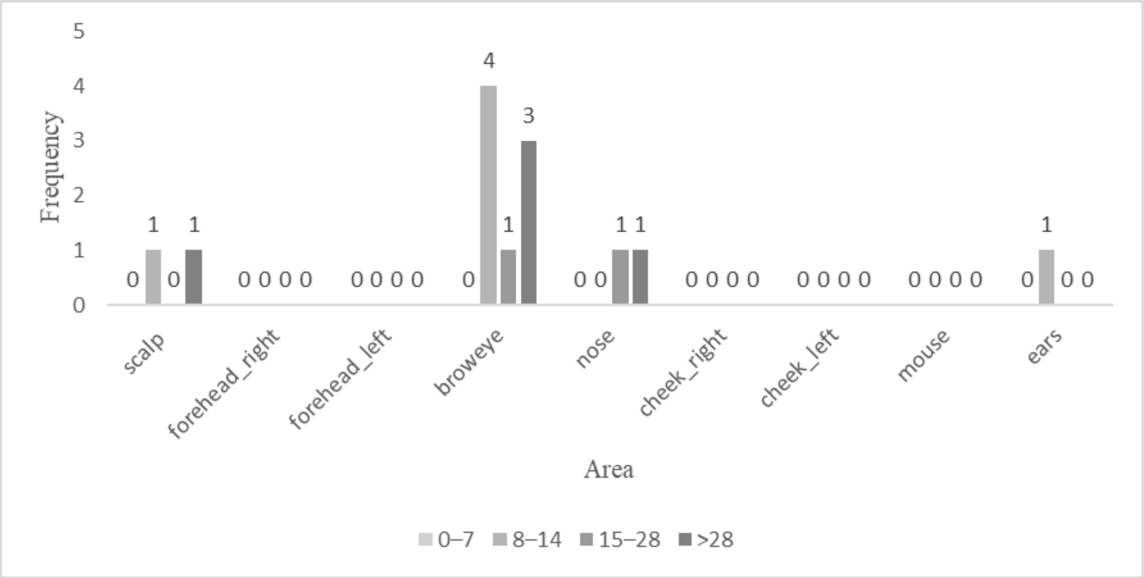

Fig S5: Relationship between number of infants who had exudate and the cure period (days). (n=10)
